# Development of methods to objectively identify time spent using active and motorised modes of travel to work: how do self-reported measures compare?

**DOI:** 10.1186/s12966-014-0116-x

**Published:** 2014-09-19

**Authors:** Jenna Panter, Silvia Costa, Alice Dalton, Andy Jones, David Ogilvie

**Affiliations:** MRC Epidemiology Unit, School of Clinical Medicine, University of Cambridge, Box 285, Cambridge Biomedical Campus, Cambridge, CB2 0QQ UK; UKCRC Centre for Diet and Activity Research (CEDAR), School of Clinical Medicine, University of Cambridge, Cambridge, UK; Norwich Medical School, University of East Anglia, Norwich, UK

**Keywords:** Physical activity, Heart rate monitoring, GPS, Convergent validity, Walking, Cycling, Transport

## Abstract

**Background:**

Active commuting may make an important contribution to population health. Accurate measures of these behaviours are required, but it is unknown how self-reported estimates compare to those derived from objective measures. We sought to develop methods for objectively deriving time spent in specific travel behaviours from a combination of locational and activity data, and to assess the convergent validity of two self-reported estimates.

**Methods:**

In 2010 and 2011, a sub-sample of participants from the Commuting and Health in Cambridge study concurrently completed objective monitoring using combined heart rate and movement sensors and global positioning system devices and reported their past-week commuting in a questionnaire (modes used, and usual time spent walking and cycling per trip) and in a day-by-day diary (all modes and durations). Automated and manual approaches were used to objectively identify total time spent using active and motorised modes. Agreement between self-reported and objectively-derived times was assessed using Lin’s concordance coefficients, Bland-Altman plots and signed-rank tests.

**Results:**

Compared to objective assessments, day-by-day diary estimates of time spent using active modes on the commute were overestimated by a mean of 1.1 minutes/trip (95% limits of agreement (LOA): −7.7 to 9.9, p < 0.001). The magnitude of overestimation was slightly larger, but not significant (p = 0.247), when walking or cycling was used alone (mean: 2.4 minutes/trip, 95% LOA: −6.8 to 11.5). Total time spent on the commute was overestimated by a mean of 1.9 minutes/trip (95% LOA: −15.3 to 19.0, p < 0.001). The mean differences between self-reported usual time and objective estimates were −1.1 minutes/trip (95% LOA: −8.7 to 6.4) for cycling and +2.4 minutes/trip (95% LOA: −10.9 to 15.7) for walking. Mean differences between usual and daily estimates of time were <1 minute/trip for both walking and cycling.

**Conclusions:**

We developed a novel method of combining objective data to identify time spent using active and motorised modes, and total time spent commuting. Compared to objectively-derived times, self-reported times spent active commuting were slightly overestimated with wide LOA, suggesting that they should be used with caution to infer aggregate weekly quantities of activity on the commute at the individual level.

**Electronic supplementary material:**

The online version of this article (doi:10.1186/s12966-014-0116-x) contains supplementary material, which is available to authorized users.

## Introduction

The concept of ‘active living’ incorporates activities across the recreational, household, transport and occupational domains [1]. As a result, interventions to promote more active lifestyles increasingly target activities performed in the course of everyday life, such as walking and cycling for transport. Accurate measurement of these activities in free-living conditions is important for understanding the descriptive epidemiology and determinants of target behaviours and behaviour change; for the evaluation of interventions; and for the attribution of subsequent health impacts.

Self-reported measures of activity can capture information on the types and contexts of behaviours, whereas objective measures can provide more accurate estimates of the intensity, frequency and duration of physical activity. Self-reported measures are often used in large studies because they may impose a lower burden on participants, are easier to administer en masse and are relatively inexpensive compared to many objective measures [2]. However, a recent review of 187 studies concluded that self-reported estimates of total physical activity generally have low correlations with those derived from objective measures [3]. Importantly, such self-report instruments tend to perform particularly poorly for activities in the transport domain [3-5]. One possible reason is that relatively simple questions are often used to assess these activities, such as asking for the usual main mode of travel to work. These fail to capture the frequency or duration of particular behaviours, which are more often asked about in relation to recreational activities. They also fail to capture day-to-day or week-to-week variation and other aspects of the complexity of travel behaviour. Whilst commuting may be habitual for many people, the total time spent walking or cycling may vary from week to week [6] and it can be difficult to disaggregate commuting from other activities with which it is often combined, such as shopping or escorting children to school [7]. Furthermore, some people use public transport, which often involves a small amount of walking and cycling [8], whilst others walk or cycle sections of a longer car journey to avoid driving in congested urban centres [9]. These combinations of active and sedentary behaviours are often not captured in simple travel behaviour questions such as those typically included in global activity questionnaires, and the quantities of physical activity associated with journeys of this kind are poorly understood [10].

As interest grows in the interface between transport and public health, researchers need valid measures of transport-related physical activity that are sensitive enough to capture the variation and complexity of travel behaviour but are also simple enough to be administered as part of longer questionnaires. However, at the time of first data collection in this study (in 2009) there were no short self-report measures of time spent walking or cycling for transport validated in free-living populations [10], and only limited validation of detailed day-by-day travel diaries had been reported [11]. In validating self-report measures in this field, the selection of a gold standard method is challenging as direct observation is often impractical. Whilst wearable cameras have been shown to be a feasible method of collecting data on mode of travel in a small convenience sample [12], it is unknown whether members of a larger, more representative population sample would consent to wearing such devices, particularly given some of the privacy-related concerns [13]. The analysis of objective physical activity data alone – even using data from multiple monitoring devices – is not sufficiently advanced to enable the time spent in different types of activities to be confidently identified [14]. On the other hand, global positioning system (GPS) devices – which are increasingly being used in research [15] – allow the objective identification of locations and journeys and therefore the computation of journey times, but provide no measure of activity or intensity other than the speed at which the wearer has moved in a given time period. Whilst several studies of travel behaviour have used GPS data, papers often report little information about the processing and cleaning of the data, which is essential information if studies are to be replicated. A recent systematic review comparing self-reported and GPS-measured journey duration found that self-reported journey times tended to be greater than those derived from GPS data, with a mean difference of 3.2 minutes/trip compared with a mean trip duration of 17.9 minutes [11]. However, this difference is not necessarily due only to error in the self-reported journey times, because the collection and interpretation of GPS data remain subject to a number of limitations including signal dropout in urban areas and potential misclassification of modes of travel [16].

It is feasible to collect data using multiple synchronous measures, and a small number of studies have used combinations of accelerometer and GPS data to identify walking, one under controlled conditions [17] and three in free-living samples [18-20]. Nevertheless, the validation of self-reported measures against those derived from hip-worn accelerometers is problematic because these devices are known to underestimate certain activities such as cycling. More accurate methods exist for estimating physical activity energy expenditure, such as combined heart rate and movement sensors which capture both heart rate and acceleration [21] but to date these have not been used to quantify time spent in specific travel behaviours in everyday life. It is therefore timely to further investigate the value of collecting synchronous location and activity data from a population sample with heterogeneous travel behaviours. This provides an opportunity to develop new methods for deriving time spent in specific travel behaviours from a combination of data sources, and to compare these objective estimates with those derived from various self-report instruments in order to clarify the strengths and limitations of detailed objective measures as well as simple and more detailed self-report instruments.

In this paper, we use data collected as part of the Commuting and Health in Cambridge study [22]: first, to develop a method for identifying time spent using active and motorised modes of transport for commuting, and total commuting travel time, from combined heart rate and movement sensors and GPS devices; and second, to compare objective and self-reported estimates of active, motorised and total commuting travel time. Specifically, we aimed to answer three research questions:

1. How do estimates of time spent using active and motorised modes for commuting, and total commuting travel time, from a detailed seven-day diary compare to those derived from objective assessment?
2. How do questionnaire estimates of usual time spent using active modes on the commute compare to those derived from objective assessment?
3. How do questionnaire estimates of usual time spent using active modes on the commute compare to those derived from a detailed seven-day travel diary?

## Methods

### Study setting and recruitment

The *Commuting and Health in Cambridge* study protocol and recruitment have been reported elsewhere [22-24]. Adults aged 16 years or older were eligible to take part in the study if they lived within 30 km of the city centre and travelled to work in Cambridge, UK. Participants were recruited in 2009 predominantly through workplaces via emails, recruitment stands and advertisements. Participants were recruited from a range of types of workplace within Cambridge, including local authorities, healthcare providers and retail outlets as well as institutions of higher and further education, and there was heterogeneity in their geographical settings which spanned city centre and urban fringe locations. Ethical approval was obtained from the Hertfordshire Research Ethics Committee (reference numbers 09/H0311/116 and 10/H0311/65) and written informed consent was provided by each participant.

### Data collection procedures

Between May and October 2009, 1164 participants completed a questionnaire to assess their physical activity, travel behaviours and personal characteristics, and a sub-sample of 475 participants who expressed a willingness to do so also wore accelerometers for seven days [23,25,26]. As the number of willing participants exceeded the number of monitors available, monitors were sent to a random sample of willing participants in batches as described in a previous paper [19]. In 2010 and 2011, the sub-sample who had completed a questionnaire and provided valid accelerometer data in 2009 were invited to wear a combined heart rate and movement sensor and a GPS device, and to complete a questionnaire and a detailed travel diary. Participants attended one-to-one appointments with a research assistant, at either the research institute or the participant’s workplace, during which the research assistant took consent and fitted the devices. Participants were instructed to wear both devices simultaneously, to complete the travel diary at the end of each day, and to complete the questionnaire at the end of the seven-day measurement period. They returned all questionnaires and devices to the research institute by post.

#### Objective measures

Participants undertook seven consecutive days of monitoring and wore combined heart rate and movement sensors and GPS devices. The Actiheart combined sensor (AccHR) is a lightweight waterproof device that clips onto two standard electrocardiogram electrodes on the chest and collects data in either 60-second epochs (using Actiheart devices in 2010) or 15-second epochs (using Actiheart 4 devices in 2011). Monitors provide no feedback to participants, are waterproof and do not need to be removed for showering, swimming or any other reason except to change electrodes if necessary. The AccHR has been shown to be a valid and reliable tool for measuring both acceleration and heart rate and offers a more accurate assessment of physical activity energy expenditure (PAEE) than accelerometry alone [21]. A simple calibration protocol based on sleeping heart rate and gender has been shown to be adequate for free-living studies [27].

A range of devices is available for collecting locational data including data loggers without displays, GPS watches and mobile phones [16]. We used the QStarz BT1000X receiver, a small, portable data logging device without a display which has been used in previous studies [28,29]. The receiver records the spatial coordinates of participants at five-second intervals. As the device is lightweight and small, it was worn on an elastic belt on the waist during waking hours and participants were provided with a charger and asked to recharge the battery each night.

#### Questionnaire and travel diary

In the questionnaire, participants reported all travel modes used on the journey to and from work on the previous seven days by completing a one-page instrument adapted from one previously shown to have acceptable test-retest reliability [30]. Participants were also asked if they ever cycled part or all of the way to work (yes or no) and if they responded ‘yes’, were then asked to report the typical duration of the cycling stage of the journey (in minutes). These questions were repeated for walking (Additional file 1). Participants concurrently completed a seven-day travel diary booklet closely based on that used in the UK National Travel Survey [31]. At the end of each day participants recorded the start and end time of each trip and the mode, distance and duration of all the stages of each journey they had made (Additional file 2). Participants also reported all the demographic and socio-economic information shown in Table 1.

**Table 1:** **Participants included for each research question**

	**RQ1**	**RQ2**	**RQ3**
	n = 42*	n = 26*	n = 102*
Sex (%)			
Male	45	42	42
Female	55	58	58
Mean age in years (SD)	46.2 (11.1)	45.4 (12.1)	45.1 (10.9)
BMI classification (%)			
Underweight/normal weight	52.4	60.0	61.6
Overweight	35.7	28.0	30.3
Obese	11.9	12.0	6.1
Education (%)			
Degree or equivalent	85.7	84.1	83.5
Less than degree	14.3	25.9	16.5
Occupation (%)			
Sedentary	76.2	72.7	77.2
Standing/manual/heavy manual work	23.8	27.3	22.8
Home ownership (%)			
Owner	71.4	80.0	81.4
Other	28.6	20.0	18.6
Home location (%)			
Urban (settlement size >10,000 inhabitants)	50.0	59.1	56.9
Town and fringe	28.6	17.1	20.6
Village and hamlet	21.4	23.8	22.5
Long standing illness (%)			
Yes	7.1	4.5	2.9
No	92.9	95.5	97.1

RQ: Research Question. Some small categories have been combined. For example, few participants were classified as underweight, and these were therefore grouped with those of normal weight.

*In RQ1 participants are not the unit of analysis, whereas for RQ2 and RQ3 participants are the units of analysis.

### Data processing

#### Objective data

**Figure 1:**
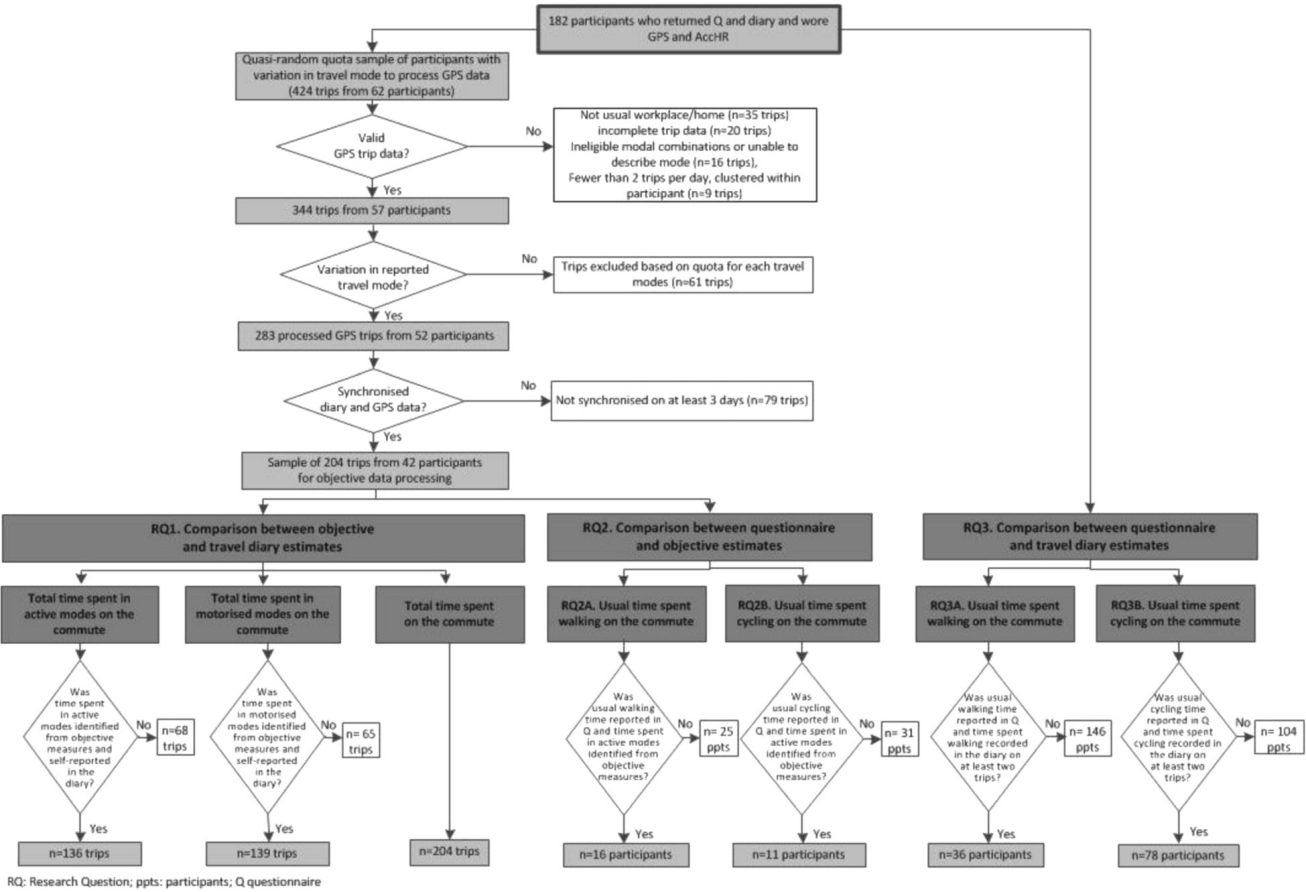
**Selection of participants for analysis and resulting sample sizes for each research question.**

To objectively identify time spent using active and motorised modes, and total commuting travel time, it was necessary to process the objective data to identify trips (or stages within trips). The manual processing and cleaning of trip-level data is a labour-intensive process, and web-based services to identify trips automatically from GPS data are in their infancy [32]. As a result, we quasi-randomly selected a purposive quota sample for analysis from the 182 participants who provided objective data and information on commuting in the travel diary and questionnaire in 2010 or 2011. This quasi-random quota sampling was designed to achieve at least 50 journeys for each of the six most commonly reported trip patterns in the sample: walking, cycling, car or motorcycle only, car in combination with walking, car in combination with cycling, and bus with or without walking or cycling. Relatively few participants reported walking all the way to or from work in the sample. This reflected our recruitment strategy for the wider study, which focussed on commuters who did not live in the same immediate area of the city as their workplace, and the relative prevalences of walking and cycling in Cambridge more generally. As a result we were unable to obtain 50 trips made by walking alone. Participants were selected for analysis if their GPS data contained at least two trips on at least one day. GPS trips were only included if they represented ‘usual’ commuting trips (i.e. between their usual home and usual work locations) and could be matched to a synchronous travel diary record on at least three days. GPS and AccHR data were summarised in one-minute epochs for the purposes of processing. Figure 1 illustrates the selection of participants for analysis.

**Figure 2:**
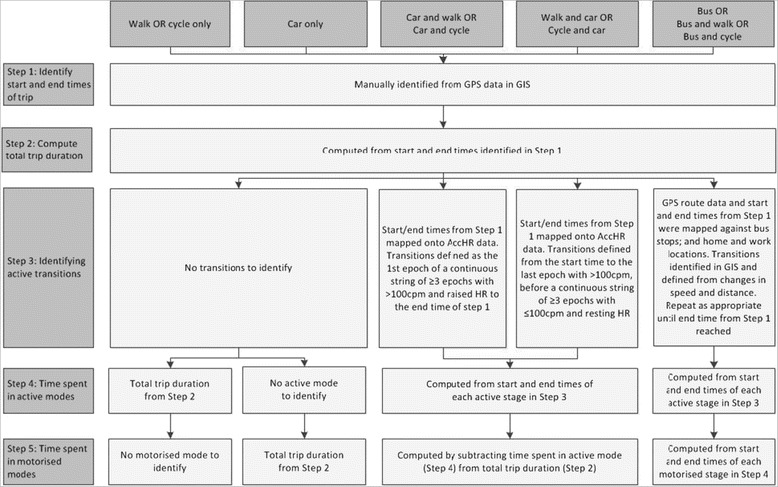
**Procedure for extracting trip-level time spent travelling and using active and motorised travel modes from combination of objective measures.**

The approach to identifying time spent using active and motorised modes, and total commuting travel time at the trip level, was tailored according to the modes of travel reported in the travel diary for that trip. Manual procedures were used in steps 1–2 and automatic procedures were used in steps 2–5. The process is described in detail below and summarised in Figure 2.

#### Step 1: Identifying the start and end times of trips

GPS data were visually inspected in the geographic information system (GIS) software package ArcGIS to identify the start and end times for each trip to and from work. Home and work locations were identified from the questionnaire and mapped using GIS and routes were overlaid with background mapping. The start time was defined as the first epoch after which participants left either the home or work location, and the end time was defined as the last epoch before the participant reached the building outline representing their workplace or home respectively.

#### Step 2: Computing total trip duration

Total trip duration was computed by subtracting the end time from the start time identified in step 1.

#### Step 3: Identifying modal transitions and destinations en route

For trips made using combinations of modes, it was necessary to identify transitions between active and motorised transport modes. As the range of intensities of activity for walking and cycling in free-living conditions is poorly understood [10], times spent in active stages of a trip were identified using GPS data with or without AccHR data as follows. For trips involving walking and cycling in combination with car travel, inspection of the AccHR data annotated with the trip start and end times from GPS indicated that the start of the active stage could be consistently defined as the first one-minute epoch of a continuous string of three or more epochs with >100 counts per minute (cpm) and a heart rate increasing at a rate of at least 10 beats per minute (bpm) per min (for example 80 to 90 to 100 bpm over three epochs). The end of the active stage was defined as the last one-minute epoch with >100 cpm before a continuous string of ≥3 epochs with <100 cpm and a heart rate decreasing at a rate of at least 10 bpm per minute (for example 100 to 90 to 80 bpm). The 100 cpm cutpoint corresponds to around 0.3 m/s^2^ acceleration and was chosen to discriminate non-walking from walking as it is one-third of the trunk acceleration associated with walking at 3 km/hr [27].

Initial inspection of the AccHR data indicated that distinguishing walking or cycling stages from bus travel within a public transport journey was difficult because there were no clear delineations in activity and heart rate, hence GPS data were mapped against public transport interchange locations to identify start and end times for each stage of a bus journey. For example, a bus journey from home to work was split into at least three stages: (1) from home to the bus stop, (2) the bus journey to the final bus stop, and (3) from the final bus stop to work, with the start and end times for each stage being visually identified from changes in the speed of travel and distance between GPS points. Waiting times at bus stops before or after boarding were identified by GPS points with non-regular speeds in close geographical proximity, and were excluded.

As not all trips were made directly between the home and work locations, we also identified whether the participant travelled to or from work via an additional location such as a school or shop (visible on background mapping) and remained there for more than five minutes without a reported change in mode. Modal transitions and destinations en route were only identified where GPS or AccHR data were of good quality and where plausible heart rate values were recorded. Where data were of insufficient quality to allow modal transitions and destinations en route to be confidently identified, trips were included only in the analysis of total trip duration (step 2) and excluded from the analyses of time spent using active or motorised modes (steps 4 and 5).

#### Step 4: Computing time spent using active modes

When walking or cycling was the only reported mode used on a trip, the time spent using that mode was equal to the total trip duration in step 2. When walking or cycling were used in combination with the car on a trip, the duration of the active stage(s) was computed either from the start time identified in step 3 to the GPS-derived end time (if the car was the first mode reported on the trip) or from the GPS-derived start time to the end time identified in step 3 (if walking or cycling was the first mode reported on the trip). For bus trips, the duration of each active stage of a trip was computed from the start and end times derived in step 3; in the case of multiple active stages, these were summed to produce the total active time per trip used in the analysis.

#### Step 5: Computing time spent using motorised modes

For bus journeys, the time spent using a motorised mode was defined using the start and end times of the bus stage identified from GPS data. When trips were reported as car-only, the total trip duration was used to define the time spent using a motorised mode. When walking or cycling were reported in combination with the car on a trip, the time spent travelling by car was calculated by subtracting the duration of the active stage(s) from the total trip duration.

#### Self-reported data

No further processing of the questionnaire data was required. For the travel diary data, the start and end times of each trip were used to compute the total trip duration and time spent in active and motorised modes were retrieved from the respective stages unless a commuting trip included a stop at a location between home and work, in which case the reported durations of each moving stage of the trip were summed to provide the total trip duration.

### Inclusion criteria

A summary of the inclusion criteria is shown in Figure 1. For research questions 1 and 2 participants with processed GPS data and synchronous travel diary data were included. For the analysis of time spent using active and motorised modes in research question 1, participants were included if they reported times using active or motorised modes. For research questions 2 and 3, participants were included only if they walked or cycled on the commute, reported a usual time in the questionnaire, and reported at least two trips involving walking or cycling in the travel diary (as it was not possible to compute mean time walking/cycling with fewer than two trips). Participants could report a usual time for both walking and cycling if they did both, and could therefore contribute to both the walking and cycling analyses. For research question 3, the availability of fully-processed GPS data was not a criterion for inclusion.

### Statistical analysis

Analyses were conducted using Stata (v.11) and SPSS (v.21) statistical software packages. Participant characteristics were summarised using means and percentages and duration and distance of trips from GPS were summarised using medians and inter-quartile ranges (IQR). All continuous variables were tested for normality using the Shapiro-Wilk test.

For the comparison between the detailed travel diary and objective estimates (research question 1), we specified our analysis at the trip level, because participants reported the time spent using each mode on each trip. As trips were clustered within participants, we tested for differences using the Newson’s within-cluster Somer’s D test [33]. A small amount of walking or cycling is often involved to get to or from the bus stop at the origin and destination, but 10 participants reported only the bus stages and failed to report their access and egress modes; their time spent walking and cycling on these trips was set to zero to reflect their reporting. For the comparison between the usual time spent walking or cycling per trip and the objective estimates (research question 2) and the mean time spent walking and cycling reported in the diary (research question 3), we specified our analysis at the participant level. Differences were tested with an adapted paired-sample Wilcoxon signed-rank test since the data were non-normally distributed.

We assessed the agreement between estimates of time using Lin’s concordance coefficient [34,35], McBride’s criteria for strength of agreement [36] and Bland-Altman plots [37]. For McBride’s criteria, concordance coefficients of <0.9 are regarded as poor, 0.90-0.95 are moderate and 0.95 to 0.99 are substantial. Post-hoc Bonferroni corrections were applied taking the multiple comparisons into account.

We hypothesised that the agreement between self-reported and objective times may differ according to whether trips were direct or indirect (via an intermediate destination), and whether they involved single modes or combinations of modes, because of the difficulties in recording times spent at intermediate destinations, waiting for public transport or in transitions between modes. We therefore stratified our analyses according to these subgroups where data were available and sample sizes were sufficient.

## Results

The sociodemographic characteristics of participants are shown in Table 1 and an overview of the sample included in each analysis is shown in Figure 1.

### Processing of GPS data

Of the 424 potential trips, 344 were complete usual commuting trips with at least three trips per participant, and of these, 204 trips (made by 42 participants who had provided matching diary data for at least three days and had reported a median of five trips/week (inter-quartile range: 3 to 7)) provided the necessary variation in travel modes and formed the dataset of GPS trips for further analysis (Figure 1).

### Research question 1: Comparison between objective and travel diary estimates

#### Total time spent using active and motorised modes on the commute

For the comparison of active travel time, 151 trips were made by active modes (or included active stages) and 136 had good quality AccHR and/or GPS data in which only one mode was used or modal transitions could be identified (Figure 1). For the comparison of motorised travel time, 155 trips were made by motorised modes (or included motorised stages) and of these, 139 had good quality AccHR and/or GPS data in which only one mode was used or modal transitions could be identified.

Time spent using active modes on the commute was overestimated in the travel diary by a mean of 1.13 minutes/trip (95% limits of agreement (LOA): −7.67 to 9.95, p = 0.001; Table 2), compared with a median total journey time of 38 minutes. The magnitude of overestimation of active travel time was slightly larger when walking or cycling was used alone (mean: 2.39 minutes/trip, 95% LOA: −6.78 to 11.55, compared with a median journey time of 17 minutes), but there was no significant difference between self-reported and objective estimates in this subset of trips (*p* = 0.247). Agreement for time spent in active modes was generally strong, as indicated by the concordance coefficients which ranged from 0.91 to 0.95. When active modes were used in combination with a motorised mode, the magnitude of the difference between estimates of active travel time was smaller (+0.40 minute/trip) but this difference remained significant (p = 0.001). Time spent using motorised modes was subject to larger overestimation than active travel time, with a mean overestimation of 10.48 minutes/trip (95% LOA: −13.62 to 34.58) compared with a median total journey time of 40 minutes. This appeared to be largely explained by trips involving motorised modes in combination with active modes (mean difference: 14.49 minutes/trip, 95% LOA: −8.86 to 37.84, compared with a median total journey time of 45 minutes.

**Table 2:** **Agreement between travel diary and objective estimates of time spent using active and motorised modes and total time spent travelling**

	**N (trips)**	**Median distance in km (IQR)**	**Median duration in minutes (IQR)**	**Lin's concordance coefficient (** ***r*** **)**	**Mean difference (** ***mean ± SD*** **)**	**95% limits of agreement**	**p-value for difference**
Time spent in active travel for trip/stage*							
All active trips	136	12.8 (7.1, 25.8)	38.0 (23.8, 54.3)	0.95	1.13 (4.49)	−7.67 to 9.95	0.001
Only walking or cycling	50	4.2 (2.2, 12.1)	17.4 (13.1, 39.1)	0.95	2.39 4.68)	−6.78 to 11.55	0.247
Walking or cycling combined with other modes	86	22.7 (12.4, 30.5)	42.4 (35.0, 61.2)	0.91	0.40 (4.23)	−7.9 to 8.69	0.001
Direct trips	121	12.1 (5.4, 24.5)	36.5 (20.0, 46.3)	0.95	1.22 (4.42)	−7.45 to 9.88	0.003
Indirect trips	15	20.8 (19.6, 41.5)	61.2 (54.4, 124.3)	0.93	0.43 (5.11)	−9.58 to 10.45	0.139
Time spent in motorised travel for trip/stage*							
All motorised trips	139	23.7 (13.2, 29.6)	40.1.0 (33.3, 58.7)	0.62	10.48 (12.30)	−13.62 to 34.58	<0.001
Car trips only	40	26.3 (20.5, 29.4)	35.0 (24.8, 45.5)	0.87	0.56 (6.04)	−11.27 to 12.40	<0.001
Car or bus trips when used in combination with other modes	99	20.8 (11.5, 32.0)	45.0 (35.7, 65.8)	0.59	14.49 (11.91)	−8.86 to 37.84	0.016
Direct trips	117	24.3 (12.5, 29.6)	38.8 (31.6, 52.8)	0.57	12.10 (12.51)	−12.41 to 36.61	0.002
Indirect trips	22	20.5 (19.6, 32.0)	60.7 (52.3, 91.0)	0.85	1.87 (6.09)	−10.07 to 13.82	<0.001
**Total time spent travelling**							
All trips	204	20.4 (8.1, 29.0)	38.3 (27.2, 54.5)	0.94	1.85 (8.75)	−15.31 to 19.01	<0.001
Single mode used	90	12.2 (4.2, 26.3)	27.8 (16.2, 42.6)	0.96	1.40 (6.05)	−10.46 to 13.26	<0.001
More than one mode used	114	23.2 (12.4, 30.5)	44.3 (36.2, 65.5)	0.90	2.21 (10.42)	−18.21 to 22.62	<0.001
Direct trips	175	19.3 (7.3, 29.0)	36.6 (24.4, 48.3)	0.89	1.74 (8.89)	−15.68 to 19.17	<0.001
Indirect trips	29	20.6 (19.6, 29.3)	64.0 (54.0, 90.9)	0.97	2.50 (7.99)	−13.1 to 18.15	0.031

*Where only one mode is used, this represents the time for the trip; where a combination of modes is used, this represents the time for the active or motorised stage(s) of the trip. Distances and durations given are derived from objective estimates. IQR: inter-quartile range.

The total number of trips included in the analysis of total trip duration (204) is greater than the sum of the numbers of ‘motorised’ (139) and ‘only walking or cycling‘ trips (50) because trips in which either modal transitions or stopovers at destinations en route could not be confidently identified were included in the analysis of total trip duration but not in the analyses of time spent using active or motorised modes. For further details please see Methods: data processing.

#### Total time spent on the commute

**Figure 3:**
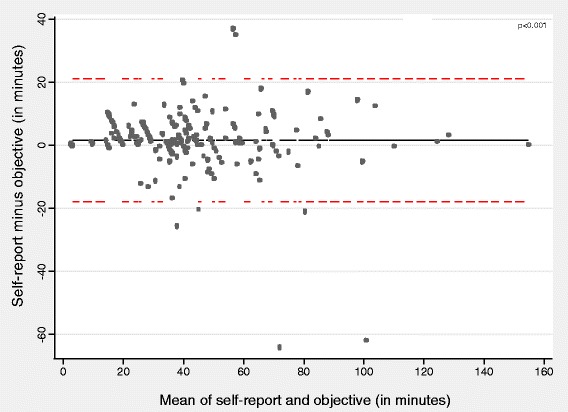
**Bland-Altman plot for agreement between travel-diary and objective estimates of total time spent travelling.** Solid lines indicate the mean difference, whilst dotted lines indicate the 95% limits of agreement.

Total travel time was overestimated in the travel diary by a mean of 1.85 minutes/trip (p < 0.001) with wide limits of agreement (95% LOA: −15.31 to 19.01; Table 2). We saw no increase in the difference between the two measures as journey duration increased (Figure 3). Agreement was closer for single-mode journeys than for those involving a combination of modes (1.40 versus 2.21 minutes/trip overestimation), although the difference between self-reported and objective estimates was significant in both trip categories (*p* < 0.001). The overestimation was also smaller for direct trips (1.74 minutes/trip, n = 175) than for indirect trips (2.50 minutes/trip, n = 29).

### Research question 2: Comparison between questionnaire and objective estimates

Of the 42 participants, 11 and 16 met the inclusion criteria for the walking and cycling analyses respectively (in that they had recorded at least two walking or cycling trips from which a mean could be computed and had reported a usual time spent walking or cycling) and provided synchronous GPS and AccHR data. For participants reporting combinations of active and motorised modes, good quality AccHR data were also required (Figure 1).

**Figure 4:**
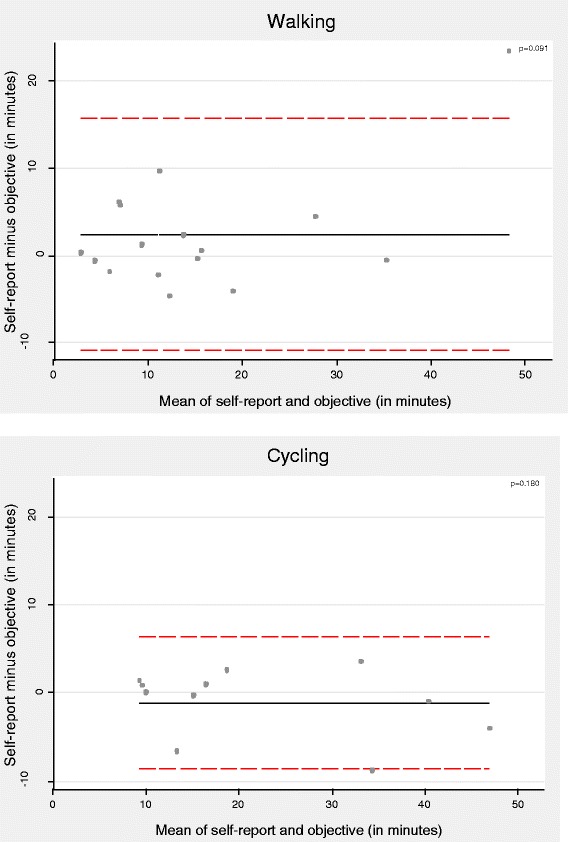
**Bland-Altman plots for agreement between questionnaire-based usual times and objectively-assessed mean times spent walking and cycling.** Solid lines indicate the mean difference, whilst dotted lines indicate the 95% limits of agreement.

The mean difference between the participants’ reported usual time and the mean time derived from the objective measures was smaller, and the 95% LOA were narrower, for cycling (mean: −1.12 minutes/trip, 95% LOA: −8.67 to 6.44, *n* = 11, compared with a median reported usual time of 17 minutes) than for walking (mean: 2.37 minutes/trip, 95% LOA: −10.91 to 15.64, compared with a median reported usual time of 13 minutes); neither difference was statistically significant (Figure 4 and Additional file 3). The questionnaire and objective measures showed substantial agreement for time spent cycling (concordance coefficient = 0.96) but poorer agreement for time spent walking (concordance coefficient = 0.84).

### Research question 3: Comparison between questionnaire and travel diary estimates

As fully-processed objective data were not required for these comparisons, the sample was drawn from the larger pool of 182 participants. Of these, 102 had synchronous questionnaire and travel diary data, reported a usual time spent walking or cycling on the commute in the questionnaire, and recorded two or more trips involving walking or cycling in the travel diary. This resulted in 36 participants for the walking analysis and 78 participants for the cycling analysis (Figure 1).

**Figure 5:**
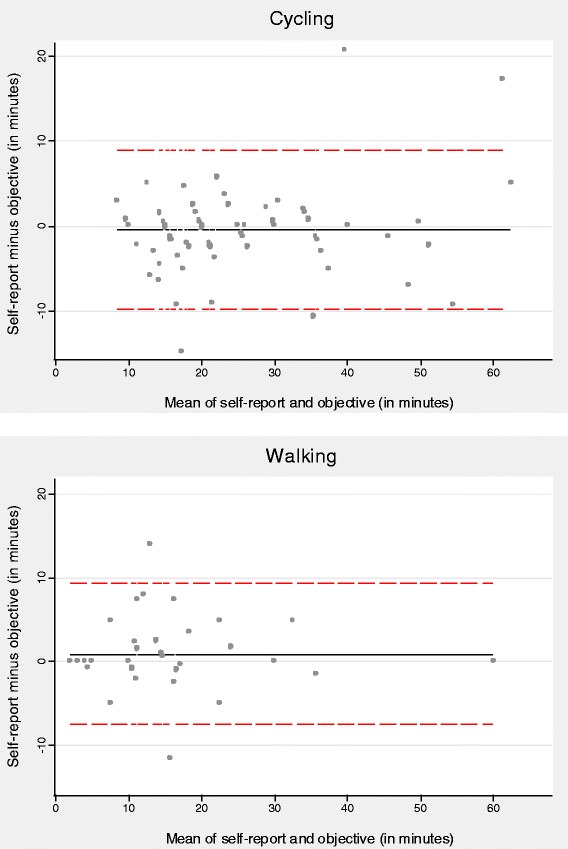
**Bland-Altman plots for agreement between questionnaire-based usual times and travel-diary mean times spent walking and cycling.** Solid lines indicate the mean difference, whilst dotted lines indicate the 95% limits of agreement.

The questionnaire estimates of usual time spent either walking or cycling were not significantly different from the mean duration derived from the trips reported in the travel diary (*p* > 0.1) and showed moderate agreement (concordance coefficients ranged from 0.92 to 0.93), with an average difference of less than one minute for both cycling and walking (Figure 5). The overestimation was slightly higher when walking was the only mode used on the trip (mean: 2.00 minutes/trip, 95% LOA: −8.65 to 12.65, compared with a median reported usual time of 20 minutes; Figure 5 and Additional file 4). Performing the same analysis excluding indirect trips did not alter the results significantly.

## Discussion

### Principal findings

In this paper we have demonstrated a method for objectively defining the time spent using active and motorised modes of transport (either alone or in combination) on the journey to work in a free-living sample using a combination of AccHR and GPS data. This was achieved by a combination of automated and manual methods and, as far as we are aware, has not previously been reported. When we compared these estimates to those derived from self-report instruments, we found the results were broadly comparable in that the mean differences in total and active travel time were relatively small, both for detailed travel diaries (around a one-minute overestimation per trip) and for retrospective questionnaires (around a one-minute underestimation for cycling and a two-minute overestimation for walking). Although these mean differences were small, self-reported measures should nevertheless be used with caution to infer aggregate weekly quantities of activity on the commute because, for example, a two-minute overestimation per trip corresponds to a 20-minute difference over a five-day working week. Self-reported time spent using motorised modes of transport showed poor agreement with objective estimates; this was mostly explained by journeys involving combinations of modes, particularly bus journeys. There were no significant differences between the usual and day-by-day estimates of time spent walking or cycling derived from the shorter questionnaire and the more detailed travel diary respectively.

### Strengths and limitations

A key strength of this study was the development of a novel method of combining detailed spatial and activity data to objectively identify the time spent using active and motorised modes and total time spent commuting. Combined heart rate and movement sensors provided more accurate estimation of activity than hip-worn accelerometers which are more often used. Our analyses of the convergent validity of objective and self-reported estimates of time investigated the differences between these measures in free-living conditions, and how these varied according to whether active or motorised modes of transport were used or combinations of modes were used, for example in public transport journeys.

We could have inferred travel mode using only the average and maximum speeds recorded on the trip, as other authors have done. However, those authors reported that this correctly identified travel mode in only 70% of cases [38]. In our sample, the risk of misclassification may have been higher as many participants travelled into city centre locations where average speeds could be relatively low, and used a combination of modes which would not have been identified. We therefore used the travel modes reported by the participants.

Our rules for processing the measurement data were newly developed for this study and remain untested in other samples. One implication of this is that the differences we observed are not necessarily wholly attributable to self-report measurement error. For example, while we collected data in one-minute epochs in 2010, in 2011 we were able to collect data in 15-second epochs as a result of technological advances. In order to combine data between waves, however, we had to summarise all the data at the one-minute level for the purposes of this analysis, and this may have contributed to the small apparent overestimation in the self-reported measures. A more general implication is that although our analysis used data from a relatively large and heterogeneous validation sample (the largest comparison containing 204 trips), our methods and findings should be replicated in larger samples. This is particularly so for the comparison of self-reported usual times with objective estimates, which was conducted at the participant level: the consequent reduction in sample size may have resulted in a lack of power to detect associations. We have sought to document our methods as clearly as possible, in the hope that other researchers will test, adapt and refine these methods in future research.

Our sample contained a large proportion (85%) of participants educated to degree level and a smaller proportion of obese adults than the general population of Cambridgeshire, no doubt reflecting the focus of the overall study on the predominantly healthy working adult population. A degree of volunteer bias is somewhat inevitable in measurement studies of this kind and it is questionable whether members of a larger, more representative population sample would be equally willing to wear multiple devices and complete multiple self-report instruments, although this possibility could be investigated in future work. We had only a few participants who reported walking all the way to work, and whilst this reflects the recruitment strategy of the overall study and local travel patterns, our smaller sample size for the analyses of time spent walking means that we can be less confident about these results than about those for the other modes of transport.

### Comparison with other studies

The magnitude of the overestimation of self-reported journey times in this study was comparable to that reported in a recent systematic review of the difference between GPS-derived and self-reported journey times, in which the pooled mean difference was 3.2 minutes or 20.3% of mean total journey time [11]. A recent study comparing self-reported journey times with those estimated using wearable cameras found that total journey time for a range of journey purposes was overestimated by around two minutes on average, with 95% limits of agreement from −9 to +13 minutes [12]. In contrast to that study, when we compared diary-based with objective estimates we did not observe an increase in the difference between the two measures as journey duration increased, suggesting that the difference was not dependent on journey length. We cannot confidently exclude the possibility of such bias with respect to self-reported estimates of usual times, because the smaller sample size for that analysis may have limited our power to detect any bias.

One of the novel aspects of this study was our assessment of the agreement between self-reported and objective estimates of time spent using active and motorised modes of transport in which we separated trips involving only one mode from those involving a combination of modes. For trips made only by walking or cycling, the difference between self-reported and objectively-derived times was not significant. However, where active and motorised modes were used in combination, the difference (while small in absolute terms) was significant. For trips made only by motorised modes, self-reported estimates were considerably less accurate with the mean overestimation increasing to around 14 minutes/trip. This probably reflects two sources of error in the self-report data, both mainly pertaining to the use of public transport. First, some participants who reported using the bus assigned their total journey time to that mode (resulting in an overestimation of the motorised travel time) and failed to report the mode of transport used to get to and from the bus stop (which was likely to have been walking). Second, participants may have included transport-related activities or public transport waiting times in their reporting of journeys in the travel diary, despite specific instructions to exclude these periods [12]. When the car was the only mode of transport reported on the journey, the mean difference was very small (less than one minute). Given the increasing research interest in time spent in sedentary behaviours such as motor vehicle use, and the uncertainty in the estimation of travel time particularly for public transport journeys, future research should aim to develop methods to elicit more accurate information about the use of motor vehicles.

### Acknowledging the likely magnitude of overestimation in self-reported measures

Objective measures, although more accurate, are not always appropriate to use in all situations. For example, self-reported methods of capturing physical activity are often more feasible for use in population surveillance. Considerable caution should be exercised in using very simple domain-specific measures of walking or cycling for transport — such as a single question to ascertain the usual main mode of travel to work — to make inferences about changes in the quantity of physical activity being undertaken [4,5]. Our more detailed and specific (but still relatively simple) self-reported measures of walking and cycling on the commute appear to have performed well, with only a small degree of overestimation on average, but they are far from ideal.

It is widely acknowledged that that the use of self-reported data may result in overestimates of time spent in physical activity. Previous studies have suggested several potential reasons for overestimation of time spent travelling using self-reported measures, such as the rounding of travel times [39] and the inclusion of visits to destinations en route and activities at either end of the journey such as parking, locking up or loading [12]. The tendency for overestimation could also reflect the way in which questions are sometimes structured such that the details and complexities of journeys are not captured. Systematic error – where such error is predictable and constant between individuals – would not be expected to introduce bias into estimates of associations with health or other outcome variables in epidemiological analyses, whereas imprecise measurement of exposure variables with random error may lead to an underestimation of such associations [40]. The error we observed in this study was non-systematic, with inconsistent evidence for patterning by journey duration. As such, we cannot predict what effect this error may have when using such measures in epidemiological studies because this would depend on the associations between errors, exposures, outcomes and other covariates in any analyses. Nevertheless, researchers should be aware of the possible cumulative effect of the overestimation of active travel time which, over a week, could be substantial and should use self-reported estimates with caution.

### Self-reported measures: fit for purpose?

Estimates of the usual time spent walking and cycling per trip were very similar to the trip-level durations reported in the detailed travel diary, and to the average durations recorded over a number of trips using a combination of objective measures. This suggests that groups of participants can accurately report such information, and because commuting is often a regular and habitual activity, it may therefore be entirely appropriate to use a simple self-reported measure if only an estimate of the usual time spent walking or cycling on the commute is required to answer the research question in a given study. The small mean overestimation of time from the self-reported measures indicates that these provide a good estimate of population levels of activity. However, the wide limits of agreement indicate a risk of substantial over- or under-estimation at an individual level. The implications of these findings depend on the research question for which such data are to be used. For population-level surveillance and monitoring, self-reported measures of time spent walking and cycling appear likely to perform well. However, for research focused on detecting changes within individuals over time, the noise of the potentially large measurement error may mask the signal of small but important real changes in behaviour. We have previously shown that the effect sizes of the most promising interventions to promote walking for transport are of the order of 15–30 minutes per week [41], which is directly comparable to the size of the measurement error suggested by the analysis reported in this paper. This suggests that evaluations of interventions that are anticipated to have relatively modest effect sizes may have limited power to detect effects on time spent in active commuting if they rely on self-reported measures alone.

## Conclusions

We have shown how a combination of data can be used to provide estimates of times spent using active and sedentary modes of transport to which self-reported estimates can be compared. In general, self-reported active, motorised and total travel times on the journey to work were subject to small overestimations at the trip level. Overestimation of travel time was smaller when active modes of transport were used alone and larger when active modes were combined with the use of motor vehicles. Self-report data should be used with caution to infer aggregate weekly quantities of physical activity attributable to commuting or to assess changes in longitudinal and evaluation studies.

## Additional files

Additional file 1:
**Questions assessing travel to work in the last seven days in the Questionnaire.**
Additional file 2:
**Example travel diary for one day.**
Additional file 3:
**Agreement between reported usual time from questionnaire and mean time derived from objective measures.**
Additional file 4:
**Agreement between reported usual time from questionnaire and mean duration reported in travel diary.**
