# Supplementary material for: Development of methods to objectively identify time spent using active and motorised modes of travel to work: how do self-reported measures compare?
Source: Int J Behav Nutr Phys Act. 2014 Sep 19;11:116. doi: 10.1186/s12966-014-0116-x (PMC4177527; doi:10.1186/s12966-014-0116-x)
Supplement: Additional file 1: — Questions assessing travel to work in the last seven days in the Questionnaire. [file 12966_2014_116_MOESM1_ESM.docx]

**Supplementary material:**

**Additional file** **1: Question assessing travel to work in the last seven days in the Questionnaire**

| **1** | Do you ever cycle part or all of the journey to or from work?This includes cycling to or from a bus stop, railway station or park-and-ride. | | | | | | | | | | |
| --- | --- | --- | --- | --- | --- | --- | --- | --- | --- | --- | --- |
|  |  | *Tick one only* | | Yes | |  | ***Go to Q.2*** | No |  | | ***Go to Q.3*** |
|  | | | | | | | | | | | |
| **2** | *How long does the cycling part of the journey usually take?* | | | | | | |  | | minutes each way | |
|  | | | | | | | | | | | |
| **3** | Do you ever walk part or all of the journey to or from work? This includes walking to or from a bus stop, railway station or park-and-ride. | | | | | | | | | | |
|  |  | | *Tick one only* | Yes |  | | ***Go to Q. 4*** | No |  | | ***Go to Q. 5*** |
|  | | | | | | | | | | | |
| **4** | *How long does the walking part of the journey usually take?* | | | | | | |  | | minutes each way | |

| **5** | In this section, we are interested in **how you travelled to and from work on each of the last seven days**.  **For each of the last seven days, please tell us what time you started and finished work and tick all the modes of transport you used on the journey to and from work.** If you did not travel to work on a particular day (either because it was a day off or because you worked at home), please tick the box ‘Did not travel to work’. If your journey to and from work was the same on more than one day, you can tick the box ‘Same as previous’ instead of repeating the information again. *We have given you an example for one day in the first row of the table.* | | | | | | | | | | | | | |
| --- | --- | --- | --- | --- | --- | --- | --- | --- | --- | --- | --- | --- | --- | --- |
| **Day  of the week** | | **Time  started  work** | **Time  finished  work** | **Did not  travel  to work** |  | **Which modes of transport did you use on this journey?** *Tick all that apply* | | | | | | | | |
|  |  |  |  |  |  | Same as previous | Guided bus | Other bus or coach | Train or  underground | Car, taxi  or van | Motorcycle or moped | Bicycle | Walking | Other |
| Thu | | 7.30 am 7.30 am | 3.30 pm |  | **To work**  **From work** |  | **✓**  **✓** |  |  |  |  |  | **✓**  **✓** |  |
|  | |  |  |  | **To work**  **From work** |  |  |  |  |  |  |  |  |  |
|  | |  |  |  | **To work**  **From work** |  |  |  |  |  |  |  |  |  |
|  | |  |  |  | **To work**  **From work** |  |  |  |  |  |  |  |  |  |
|  | |  |  |  | **To work**  **From work** |  |  |  |  |  |  |  |  |  |
|  | |  |  |  | **To work**  **From work** |  |  |  |  |  |  |  |  |  |
|  | |  |  |  | **To work**  **From work** |  |  |  |  |  |  |  |  |  |
|  | |  |  |  | **To work**  **From work** |  |  |  |  |  |  |  |  |  |
