# Supplementary material for: Development of methods to objectively identify time spent using active and motorised modes of travel to work: how do self-reported measures compare?
Source: Int J Behav Nutr Phys Act. 2014 Sep 19;11:116. doi: 10.1186/s12966-014-0116-x (PMC4177527; doi:10.1186/s12966-014-0116-x)
Supplement: Additional file 3: — Agreement between reported usual time from questionnaire and mean time derived from objective measures. [file 12966_2014_116_MOESM3_ESM.docx]

Additional file 3 - Agreement between reported usual time from questionnaire and mean time derived from objective measures

|  | **All cycling trips** | **All walking trips** |
| --- | --- | --- |
| Sample of participants (*n*) | 11 | 16 |
| **Median reported duration in min**utes **(IQR)** | **17 (10, 35)** | **12.5 (10, 16.5)** |
| Lin's Concordance Coefficient (*r*) | 0.96 | 0.84 |
| Mean difference in min (*SD*) | -1.12 (3.85) (3.85) | 2.37 (6.77) |
| 95% LOA | -8.67 to 6.44 | -10.91 to 15.64 |
| Wilcoxon sign-rank (*ρ*) | 0.657 | 0.352 |

LOA: Limits of Agreement. Median durations given are derived reported usual time from questionnaire
