# Supplementary material for: Development of methods to objectively identify time spent using active and motorised modes of travel to work: how do self-reported measures compare?
Source: Int J Behav Nutr Phys Act. 2014 Sep 19;11:116. doi: 10.1186/s12966-014-0116-x (PMC4177527; doi:10.1186/s12966-014-0116-x)
Supplement: Additional file 4: — Agreement between reported usual time from questionnaire and mean duration reported in travel diary. [file 12966_2014_116_MOESM4_ESM.docx]

Additional file 4 - Agreement between reported usual time from questionnaire and mean duration reported in travel diary

|  | **Cycling** | | | **Walking** | | |
| --- | --- | --- | --- | --- | --- | --- |
|  | **All trips** | **Single mode** | **Combination of modes** | **All trips** | **Single mode** | **Combination of modes** |
| Sample (*n*) | 78 | 59 | 19 | 36 | 9 | 27 |
| **Median reported duration in min**utes **(IQR)** | **20 (15, 30)** | **20 (17, 35)** | **15 (10, 25)** | **12 (10, 20)** | **20 (16, 30)** | **10 (10, 15)** |
| Lin's Concordance Coefficient (*r*) | 0.93 | 0.93 | 0.85 | 0.92 | 0.95 | 0.84 |
| Mean difference in min (SD) | -0.53 (4.76) | -0.48 (4.90) | -0.70 (4.44) | 0.94 (4.30) | 2.00 (5.43) | 0.59 (3.91) |
| 95% LOA | -9.86, 8.80 | -10.07, 9.12 | -9.40, 8.00 | -7.49, 9.37 | -8.65, 12.65 | -7.08, 8.25 |
| Wilcoxon sign-rank (*ρ*) | 0.108 | 0.127 | 0.572 | 0.178 | 0.336 | 0.322 |

LOA: Limits of Agreement. Median durations given are derived from reported usual time from questionnaire.
